# COVID-19, mental health and psychological first aid

**DOI:** 10.1017/ipm.2020.41

**Published:** 2020-05-14

**Authors:** E. Minihan, B. Gavin, B. D. Kelly, F. McNicholas

**Affiliations:** 1 School of Medicine, University College Dublin, Belfield, Dublin 4, Ireland; 2 Department of Psychiatry, Trinity College Dublin, Trinity Centre for Health Sciences, Tallaght University Hospital, Dublin 24 D24 NR0A, Ireland

**Keywords:** COVID-19, coronavirus, crisis, mental health, pandemic, psychological first aid, stress

## Abstract

Crises such as the global pandemic of COVID-19 (coronavirus) elicit a range of responses from individuals and societies adversely affecting physical and emotional well-being. This article provides an overview of factors elicited in response to COVID-19 and their impact on immunity, physical health, mental health and well-being. Certain groups, such as individuals with mental illness, are especially vulnerable, so it is important to maximise the supports available to this population and their families during the pandemic. More broadly, the World Health Organization recommends ‘Psychological First Aid’ as a useful technique that can help many people in a time of crisis.

## The effects of stress

Stress, the body’s reaction to real or perceived harmful situations, has been evoked at both an individual and societal level as a response to COVID-19. While acute stress reactions might be protective, when extreme and long-lasting and when viewed as outside of our control, prolonged stress may lead to longer term pathology. There are a number of particular features relating to the current pandemic that make it reasonable to assert, based on the current aetiological understanding of stress and anxiogenic factors, that COVID-19 is particularly likely to result in increased psychological and psychiatric morbidity. Not least of these are the huge knowledge gap pertaining to the scientific understanding of the virus (and by extension that of the general public), its markedly variable course and prognosis, and the absence of certainty as to how this situation will evolve, as well as its pernicious effect. Given the infection control protocols pertaining in this pandemic, patients’ families may experience particularly high levels of stress due to restrictions on visitation and face-to-face clinical discussions with treating clinicians, and fear induced by the donning of personal protective equipment, together with the fear of contracting the virus and passing it on to other family members. For families who are bereaved, grief is likely to be complicated by the inability to engage fully in traditional funeral rituals with family and friends. While stress, in certain contexts, in small doses and for short periods can be beneficial in allowing one to cope with the stressor, prolonged stress has negative effects on both physical and mental health. There is a causal relationship between stressful life events and major depressive episodes, with individuals being 2.5 times more likely to be depressed patients compared to controls (Hammen, 2005), and an increased risk of admission for depression (Kessing *et al.* 2003). Anxiety, too, is often linked with stressful events and commonly occurs before depression (Schneiderman *et al.* 2005). -Stressful events may promote behaviours that are harmful and cause further stress, such as smoking, increased alcohol consumption, sleep problems and disordered eating.

The theoretical framework underpinning societal stress responses is based on ‘contagion theory’ (LeBon, 1897) which describes collective behaviours in groups (Khan & Huremović, 2019). This model has been developed to incorporate the impact of a crowd on an individual’s emotional and behavioural response (Park and Burgess, 1921). The term ‘emotional contagion’ is used to describe the spread of affect and mood through populations simply through people’s exposure to each other, while ‘behavioural contagion’ is the spontaneous imitation of a crowd’s behaviours. Different elements of these ‘contagion’ effects are evident during the current pandemic such as panic buying or donning of homemade masks. Within this model, it is postulated that anxiety and panic spread through populations owing chiefly to the anxieties of others (Saner, 2020). This is termed as an ‘emotional epidemic’, such as that accompanying the 2009 H1N1 pandemic (Ofri, 2009). While this component of the societal pandemic stress response was reported to dissipate quickly, the impact of individual stress responses had more enduring sequelae (Ofri, 2009).

Individuals in crisis situations or during disasters commonly experience enduring psychological trauma which develops into depression, anxiety disorders or substance abuse (Schneiderman *et al.* 2005). Loss of income, perceived threat to life and personal injury are all associated with mental illness. Up to one in five survivors of the 2013–2016 Ebola epidemic experienced post-trauma reactions similar to those seen in victims of more typical traumatic events such as armed conflict, natural disasters and personal assault (Hugo *et al.* 2015). Healthcare workers are not immune to the stressogenic effect, and following the 2002–2004 outbreak of Severe Acute Respiratory Syndrome (SARS), clinical distress was detected in one-third to a half of healthcare workers at the time, with elevated levels still evident 2 years later (Maunder *et al.* 2008). The management of such pandemics, such as isolation and quarantine, also contribute to stress, with individuals reporting despair, fear, loneliness, extreme boredom and anger, with some taking their own life (Brooks *et al.* 2020). The longer the isolation, the more susceptible the person is to serious depressive symptoms (Khan & Huremovic, 2019). The psychological effects of quarantine therefore should not be underestimated (Brooks *et al.* 2020).

Stressors provoke a range of emotions and psychological reactions including, most notably, fear (Khan & Huremović, 2019). In some cases, fear can be productive and can act as a motivating factor for people to take action and come together as a society to ensure safety and order. Alternatively, fear can have a negative effect and can inhibit people from taking necessary actions. There are, for example, reports from previous outbreaks of healthcare staff trying to flee and refusing to treat patients (Barbisch *et al.* 2015). Fear can also make people react in inappropriate ways to try to avoid the threat, especially if ill-informed (US Department of Health and Human Services, 2019). Denial, the refusal to acknowledge imminent harm or that harm has already occurred, is another common reaction during an acute crisis (US Department of Health and Human Services, 2019). Stigmatisation is another common, unhelpful psychological reaction to events such as outbreaks of infectious disease. US Latinos, for example, were stigmatised during the H1N1 pandemic (McCauley *et al.* 2013). Some of the earliest cases of the outbreak were reported near a Mexican pig farm and this led to stigmatisation of people from Mexico owing to this association. Stigmatisation often stems from fear. During the SARS outbreak, the Chinese population was scapegoated by the media and unfairly apportioned blame (Eichelberger, 2007). Evidence of this stigmatisation and discrimination is present in this pandemic, with persons at high risk feeling stigmatised for using resources (Ryan *et al.* 2020) or different ethnic groups who were blamed for the origins of the disease (Ren *et al.* 2020).

The human immune system is significantly affected by stress and stressful events (Glaser & Kiecolt-Glaser, 2005), contributing to chronic fatigue, depression and immune disorders (American Psychological Association, 2006). Furthermore, depression, isolation and loneliness all affect the immune system and all are relevant during the present pandemic. In addition, psychological distress alters immune responses to the influenza vaccine (Seiler *et al.* 2020) and results in a lower antibody response to vaccination in general (Segerstrom *et al.* 2012; Pedersen *et al.* 2009). This might well prove a further complicating factor later in the current pandemic, if a vaccine for COVID-19 is eventually identified. Overall, stress can not only weaken our immune system, but also affect our response to vaccination.

Stress can also present as respiratory problems, such as shortness of breath and rapid breathing, increased oxygen demand (Yaribeygi *et al.* 2017) and place those with pre-existing respiratory disease, such as asthma and chronic obstructive pulmonary disease, at most risk (Roche *et al.* 2013). In addition, anxiety and depression are both associated with an increase in asthma symptoms (Richardson *et al.* 2006). This intensification of respiratory symptoms owing to stress can, in turn, further exacerbate anxiety, leading people to consider consulting their general practitioner or attending an emergency department. This can cause yet further anxiety and complexity during the current pandemic as many people are reluctant to attend medical services in the first place and those who do worry that their symptoms might be similar to those of COVID-19 (World Health Organization, 2020). This presents challenges for patients as well as hard-pressed staff in primary care and hospital settings who must ensure that patients with new or exacerbations of pre-existing respiratory symptoms are fully investigated and are distinguished from those with anxiety-related respiratory difficulties, requiring a different treatment approach.

Similar scenarios relating to cardiac issues are also likely during the pandemic given the association of stress and hypertension and cardiovascular disease. Stress activates the sympathetic nervous system, increases heart rate and causes narrowing of veins (Yaribeygi *et al.* 2017). Stress can also activate the parasympathetic nervous system and result in a decreased heart rate, as well as sustained muscle tension, resulting in tension headaches, migraines and lower back problems. As a consequence, people in crisis situations tend to have apparently unexplained physical symptoms, such as headaches, muscle aches and stomach upset, that are primarily due to stress (US Department of Health and Human Services, 2019). Again, some of these symptoms can resemble those of COVID-19, which can present with fever, headaches and muscle pain (Cascella *et al.* 2020).

## High-risk groups and maximising supports

Outbreaks such as SARS and Ebola had substantial psychological impacts on healthcare workers not least because many felt that they were treated like prisoners due to restrictions put in place (Barbisch *et al.* 2015). Levels of dedication dropped and some staff refused to provide care to patients (Barbisch *et al.* 2015). Providing care and communicating with patients through several layers of personal protective equipment add another layer of complexity to work in healthcare settings, along with difficult decisions about the allocation of scare resources such as ventilators or beds in intensive care units. The ‘moral trauma’ associated with these decisions might well have long-term repercussions for some staff (Maunder *et al.* 2008).

People with pre-existing mental health disorders are especially vulnerable during times of crisis (Kelly, 2020). Patients in isolation or quarantine with pre-existing illnesses should continue treatment for their psychological or psychiatric problems (Khan & Huremović, 2019). It is important to ensure that psychiatric medication does not interact with any additional medication for the infectious disease and that all prescribed medications are clearly justified. Drug interactions can often be a cause for concern, and concurrent drug administration can cause increased toxicity of the compounds (Spina *et al.* 2003).

Patients with substance use disorders might require urgent detoxification before being isolated or quarantined. People detoxing from alcohol for example can experience anxiety, fever, tachycardia and hallucinations, among other distressing symptoms. It is important that these symptoms are controlled if the person is to be isolated or quarantined.

Patients with cognitive disorders, cognitive impairment or learning difficulties might be unable to be quarantined alone or to follow the necessary instructions. In some cases, these patients are unable to care for themselves and unable to understand fully the crisis situation. It is important that the person understands what is happening as best they can and be offered support if in isolation, in order to minimise their anxiety.

People with depression and anxiety are particularly vulnerable in times of crisis, especially if they need to be isolated or quarantined. Common symptoms include low mood, trouble sleeping and feelings of guilt or worthlessness (Parekh, 2017). It is useful to counteract these symptoms using supportive therapy, reassurance, accurate information and treatment for depression or anxiety. Provision of accurate information is key to reduce the sense of uncertainty and panic and increase life satisfaction (Dulmus & Hilarski, 2003).

Maintaining communication with family and friends is critical during isolation, and if it is not possible for the person to be in direct contact with family or friends, then healthcare professionals should try to provide a sense of support and communication.

## Psychological first aid

Psychological first aid (PFA) is defined as a ‘humane, supportive response to a fellow human being who is suffering and who may need support’ (World Health Organization, War Trauma Foundation and World Vision International, 2011). The PFA model requires the provision of immediate help and support to individuals who are experiencing/have experienced distress due to a recent crisis. While it is not intended as a long-term solution, this method of care is valuable and timely during an emergency, such as the current COVID-19 pandemic. PFA has been devised as an approach and a tool that may be provided by all, not just healthcare professionals. It allows mobilisation of societal resources at a time when healthcare professionals might be needed for other tasks. Consequently, PFA is not professional counselling, and practitioners should be aware of the limitations of this method and evoke additional professional support when required. To this end, the model identifies key signs that might indicate a need for more professional involvement, such as possible harm to self or others, long-lasting or severe distress or an inability to function in daily life.

Despite the limitations, key outcomes of PFA, such as feeling safe, connected, calm and hopeful, are reported as effective in helping long-term recovery following a crisis, even when delivered by individuals without professional mental health training (Fox *et al.*, 2012). The extant literature suggests PFA has widespread appeal and has been safely administered by a range of non-professionals in a range of settings (Fox *et al.* 2012).

PFA is simple and straightforward, focused on methods that everyone can use to help reduce distress in a time of fear, anxiety and uncertainty. PFA should be carried out in a private setting that facilitates confidentiality and the safety to speak openly if the person wishes. Key features of PFA include being supportive but non-intrusive, recognising that people having the right to accept or decline assistance. PFA also involves active listening but without applying pressure to speak if the person does not feel comfortable doing so. It offers comfort and supports calmness during times of crisis. It is also important to protect the individual from further harm, including further psychological distress or risk of infection.

A step by step approach is offered in The Psychological First Aid Guide for field workers commencing with establishing basic needs, such as access to adequate supplies of food and water, especially if access to shops and other resources is limited (World Health Organization, War Trauma Foundation and World Vision International, 2011). Following this, it is essential to learn if the individual has specific new or pre-existing healthcare needs and link them to the appropriate help available. Ensure those who may be particularly vulnerable are not overlooked, such as the inform, the elderly, the young and those with mobility or communication issues who do not self-present. Given that it is likely that, post-disaster, the individual may have many needs, it is necessary to prioritise most urgent needs first. Individuals providing PFA can reinforce positive coping mechanisms and discourage negative coping strategies. When possible, people should be linked with loved ones and means of contact made available because access to social support networks augments coping.

## Conclusion

PFA, is a tried and tested model often used and recommended at times of disaster (The American Red Cross, The American Psychological Association and The United Nations). It is a simple effective way for first aiders, to help others at times when psychological distress is likely to be high. This approach provides support and comfort in times of crisis, paying attention to immediate basic needs, followed by limiting the negative effects of stress and optimising access to specialist care if needed and promotes better mental health and individual well-being. It is a simple, cost-effective step that can make a big difference in difficult, challenging times and has application in a variety of social and healthcare community and residential settings. This method of support may be delivered by existing staff and may reduce fear and isolation, promote coping and resilience and thereby offset the predicted increase in psychological and psychiatric morbidity post-pandemic. Basic training in PFA is warranted to prepare for other inevitable disasters, ensure effectiveness and reduce the risk of any adverse outcomes (Everly & Lating, 2017).
